# Determining Gut Microbial Dysbiosis: a Review of Applied Indexes for Assessment of Intestinal Microbiota Imbalances

**DOI:** 10.1128/AEM.00395-21

**Published:** 2021-05-11

**Authors:** Shaodong Wei, Martin Iain Bahl, Simon Mark Dahl Baunwall, Christian Lodberg Hvas, Tine Rask Licht

**Affiliations:** a National Food Institute, Technical University of Denmark, Kgs Lyngby, Denmark; b Department of Hepatology and Gastroenterology, Aarhus University Hospital, Aarhus, Denmark; University of Bayreuth

**Keywords:** dysbiosis, dysbiosis index, gut, imbalance, intestine, microbiome, microbiota

## Abstract

Assessing “dysbiosis” in intestinal microbial communities is increasingly considered a routine analysis in microbiota studies, and it has added relevant information to the prediction and characterization of diseases and other adverse conditions. However, dysbiosis is not a well-defined condition. A variety of different dysbiosis indexes have been suggested and applied, but their underlying methodologies, as well as the cohorts and conditions for which they have been developed, differ considerably. To date, no comprehensive overview and comparison of all the different methodologies and applications of such indexes is available. Here, we list all types of dysbiosis indexes identified in the literature, introduce their methodology, group them into categories, and discuss their potential descriptive and clinical applications as well as their limitations. Thus, our focus is not on the implications of dysbiosis for disease but on the methodological approaches available to determine and quantify this condition.

## Abstract (precis)

Assessing “dysbiosis” in intestinal microbial communities is increasingly considered a routine analysis in microbiota studies, and it has added relevant information to the prediction and characterization of diseases and other adverse conditions. However, dysbiosis is not a well-defined condition.

## INTRODUCTION

## ASSESSMENT OF DYSBIOSIS

Imbalance, dysfunction, or disturbance of the gut microbiota is increasingly recognized as an indicator of a given disease or a poor health status. Due to the complexity and huge interindividual variation in the microbial communities, no gold standard exists to determine the presence or extent of a given imbalance or disturbance, although a multitude of studies refer to it broadly with the term “dysbiosis.” An important part of the challenge with defining dysbiosis arises from the fact that due to the huge interindividual variation existing in the healthy population, no clear definition of a healthy gut microbiota has been established to date.

Dysbiosis of the gut microbiota has been associated with numerous adverse conditions, such as Clostridioides difficile (formerly designated Clostridium difficile) infection (CDI) (1), metabolic syndrome (2, 3), inflammatory bowel disease (IBD) (4), colorectal cancer (5), chronic hepatitis (6), common variable immunodeficiency (7), and even schizophrenia (8). Dysbiosis has also been observed in nonintestinal microbial communities, such as those of the gums (9), oral mucosa and saliva (10), and scalp and forehead (11). The application of the term dysbiosis is quite broad, ranging from a change of a single species to the perturbation of entire microbial communities (12).

To qualify the term dysbiosis, several indexes have been defined and applied. Such indexes may help to characterize diseases and adverse conditions, predict treatment outcomes, and provide information other than the commonly used alpha and beta diversity assessments. It is worth noting that some researchers have applied the term dysbiosis even to conditions associated with improved health status, e.g., as a result of metformin treatment against diabetes mellitus type 2 (13). However, since the prefix “dys” (Greek for “bad,” “difficult,” or “disordered”) implies an adverse condition, in the current context, we will refer to dysbiosis as a condition differing from the normal or healthy state. It should, however, be highlighted that due to a significant interindividual variation, defining the normal or healthy gut microbiota remains an inherent challenge (14); consequently, most dysbiosis indexes are based on comparison to a set of individuals or samples used as references. Even so, it is important to emphasize that dysbiosis is not a well-defined condition, and that dysbiosis indexes differ with respect to methodology and clinical context and were developed in different cohorts of individuals to describe a variety of different conditions.

Basic methods and principles related to microbiota assessment in a clinical context were recently reviewed (15). In the present review, we provide a summary of currently applied dysbiosis indexes and explain their calculation and performance in the context of specific diseases and conditions. We consider this very relevant for the choice and use of dysbiosis indexes in future studies. However, the potential causal links between intestinal dysbiosis and human health are not captured by any of the dysbiosis indexes and, thus, are outside the scope of this review.

## TYPES OF DYSBIOSIS INDEXES

In May 2020, we searched the scientific literature indexed in the PubMed database for the various combinations of the search terms “dysbiosis” or “disruption” and “score” or “index,” together with “gut” or “intestine,” in all search fields. We assessed all previous studies regarding the definition and application of gut dysbiosis indexes appearing in these searches.

**FIG 1:**
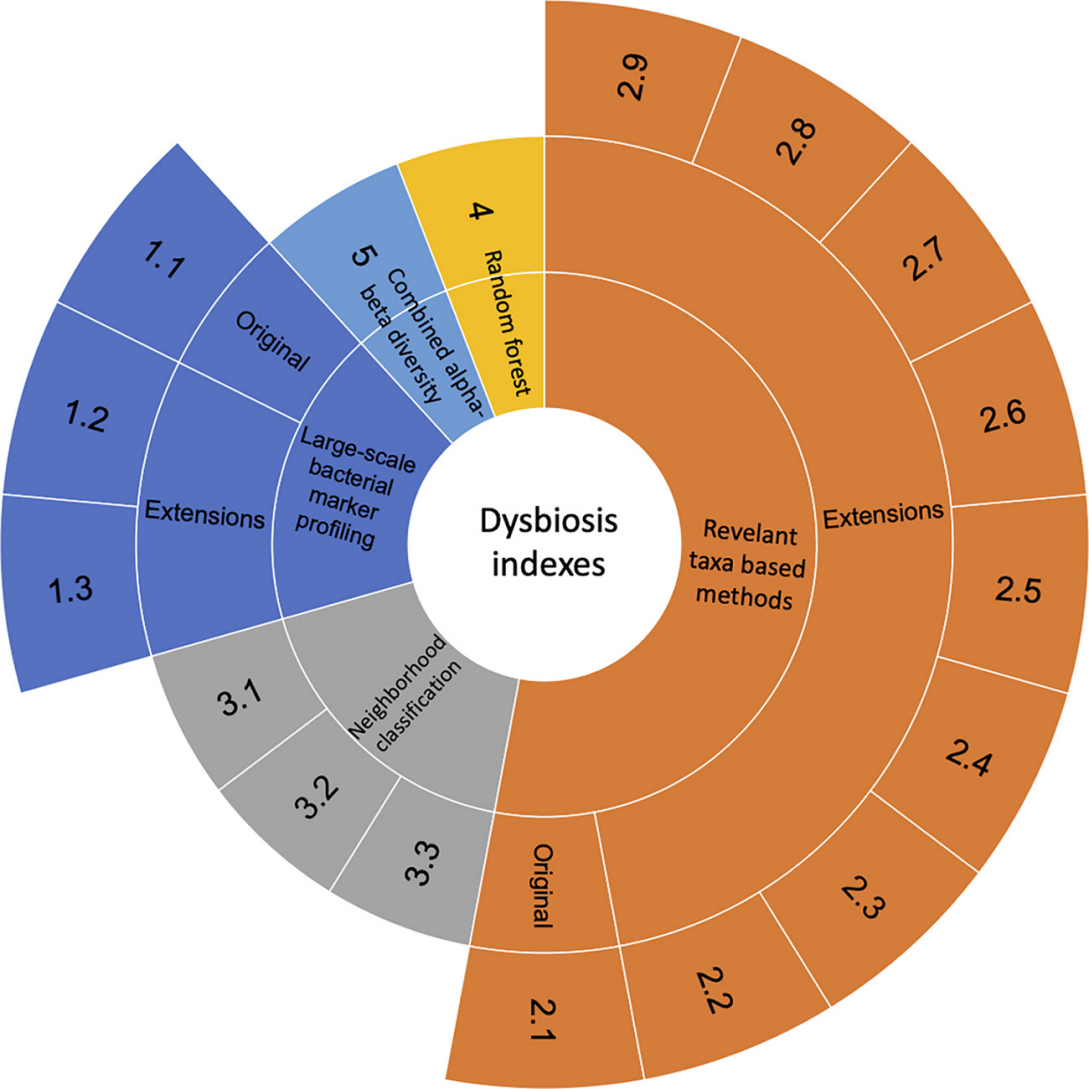
Hierarchical chart to show different indexes used to characterize the dysbiosis of microbial communities. Numbers showing in the outmost layer refer to different indexes. Original corresponds to indexes that are defined early in time and used prevalently. Extension indicates the indexes that are either derived or generalized from the original indexes.

We grouped the identified dysbiosis indexes into five categories based on the methodology, including large-scale bacterial marker profiling, relevant taxon-based methods, neighborhood classification, random forest prediction, and combined alpha-beta diversity (Fig. 1). The generalizations and extensions of a given method were described when possible. A detailed overview of the different indexes was compiled (Table 1). Below, we introduce the five categories of indexes, starting from the most prevalently used.

**TABLE 1:** Different indexes to characterize the dysbiosis of microbial communities

Dysbiosis index	Method type	Description	Disease/condition	Method	Reference(s)
1.1	Large-scale bacterial marker profiling	A set of 54 probes targeting 16S RNA gene (V3–V7) and covering more than 300 bacterial markers; dysbiosis index score calculation is a commercial secret, and scores range from 1 to 5 (2 indicates dysbiosis); scores of each taxon range from −3 to 3, where a negative value refers to reduced abundance compared to the reference population	IBS, IBD	Microarray	16
1.2	Modified large-scale bacterial marker profiling	Summed GA map score of 7 taxa selected to differ between metformin and NNS treatment (*Alistipes*, *Proteobacteria*, *Shigella* spp., *Escherichia* spp., Bacteroides fragilis, Ruminococcus gnavus, *Bacteroides* spp., *Prevotella* spp., and Dialister invisus); the index is scaled from −14 to 14	NNSs versus metformin	Microarray	13
1.3	Modified large-scale bacterial marker profiling	Median bacterial scores for 10 taxa differing between responders and the nonresponders (higher abundance of Bacteroides fragilis, Acinetobacter, *Ruminiclostridium*, *Streptococcus*, and *Eubacterium* in responders; higher abundance of *Clostridia*/*Negativicutes*/*Bacilli*, *Actinomycetales*, *Anaerotruncus*, *Clostridiales*, and *Shigella/Escherichia* in nonresponders) are defined as the cutoff; each sample is given a point for each differential taxon; the points for each sample are then summed up, resulting in an index between 0 and 10	FODMAPs diet for patients with IBS	Microarray	22
2.1	Relevant taxon-based methods	Dysbiosis was calculated as log*_e_* of (summed abundance of taxa increased in patients with CD/summed abundance of taxa decreased in patients with CD)	CD	16S ribosomal amplicon sequencing and shotgun metagenomics	32
2.2	Relevant taxon-based methods	Dysbiosis was calculated as (summed abundance of taxa increased in patients with cirrhosis/summed abundance of taxa decreased in patients with cirrhosis)	Cirrhosis	Multitag pyrosequencing of 16S genes	34
2.3	Relevant taxon-based methods	Dysbiosis was calculated as (summed abundance of taxa increased in patients with CHB/no. of CHB-increased taxa) − (summed abundance of taxa increased in subjects without CHB/no. of taxa increased in the absence of CHB)	CHB	16S ribosomal amplicon sequencing	6
2.4	Relevant taxon-based methods	Dysbiosis was calculated as [(summed abundance of taxa increased in patients with stroke/no. of stroke-increased taxa) − (summed abundance of taxa increased in subjects without stroke/no. of taxa increased in the absence of stroke)]×100	Stroke	16S ribosomal amplicon sequencing	35
2.5	Relevant taxon-based methods	Dysbiosis was calculated as [(summed abundance of taxa increased in patents with gout/no. of gout-increased taxa) − (summed abundance of taxa increased in subjects without gout/no. of taxa increased in the absence of gout)] × 1,000,000	Gout	16S ribosomal amplicon sequencing	36
2.6	Relevant taxon-based methods	Dysbiosis was calculated as log_10_[(summed abundance of taxa increased in case 1/summed abundance of taxa decreased in case 1) × (summed abundance of taxa increased in case 2/summed abundance of taxa decreased in case 2) × (summed abundance of taxa increased in case 3/summed abundance of taxa decreased in case 3) × (summed abundance of taxa increased in case 4/summed abundance taxa decreased in case 4) + 1]; case 1, 2, 3, and 4 refer to carcinoma, carcinoma adjacent, adenoma, and adenoma adjacent	Colorectal cancer (tumorigenesis)	16S ribosomal amplicon sequencing	5
2.7	Relevant taxon-based methods	Acinetobacter johnsonii and Streptococcus salivarius were either positively or negatively associated with RAS; dysbiosis was calculated as 5.35 × (abundance of *A. johnsonii*) − 0.309 × (abundance of *S. salivarius*)	RAS	16S ribosomal amplicon sequencing	10
2.8	Relevant taxon-based methods	Dysbiosis was calculated as (abundance of *Firmicutes*)/(abundance of *Bacteroidetes*)	LC, HF, IBS	16S ribosomal amplicon sequencing	37–39
2.9	Relevant taxon-based methods	Dysbiosis was calculated as (abundance of *Basidiomycota*)/(abundance of *Ascomycota*)	IBD	16S ribosomal amplicon sequencing and ITS2	42
3.1	Neighborhood classification	Dysbiosis index was calculated as the median Bray-Curtis distance between the test sample and the reference	UC, CD	Shotgun metagenomics	43
3.2	Neighborhood classification	Seven selected taxa were reported to be associated with CE in dogs; their abundances were determined by qPCR; dysbiosis was calculated as the difference between (Euclidean distance between the test sample and the healthy class centroid) and (Euclidean distance between the test sample and the diseased class centroid)	CE	qPCR	46
3.3	Neighborhood classification	Dysbiosis was calculated as log_2_-transformed CLOUD statistic	SIBO	16S ribosomal amplicon sequencing	54, 55
4	Random forest prediction	Dysbiosis was calculated as out-of-bag probability of random forest to differentiate patients with SIBO from healthy patients	SIBO	16S ribosomal amplicon sequencing	55
5	Combined alpha-beta diversity	Dysbiosis was calculated as (mean difference of Shannon diversity between the test sample and each of donors) × (mean Jensen-Shannon divergence of the test sample and each donor)	CDI	16S ribosomal amplicon sequencing	1

### Category 1: large-scale bacterial marker profiling.

Large-scale profiling of bacterial markers determines the dysbiosis of the gut microbiota by simultaneously identifying a large number of carefully selected marker species or taxa. This type of dysbiosis determination is exemplified by the GA-map dysbiosis test (Genetic Analysis AS, Oslo, Norway), which is one of a few commercial products designed to determine and characterize the dysbiosis of gut microbiota (16). The technology of this product is based on a set of 54 probes targeting the 16S rRNA gene (V3 to V7) at different bacterial taxonomic levels, thereby covering six phyla, *Firmicutes, Proteobacteria, Bacteroidetes, Actinobacteria, Tenericutes,* and *Verrucomicrobia*, which includes 10 bacterial classes, 36 genera, and more than 300 species (Fig. 1, index 1.1). The GA-map dysbiosis test assigns each sample a score from 1 to 5, where a score greater than 2 indicates that the microbial profile is different from a selected reference population and, thus, designated “dysbiosis,” while a score of 2 or lower is defined as “normobiosis,” indicating a healthy state. Additionally, the species targeted by the test are scored from −3 to 3, where a negative value refers to reduced abundance and a positive value indicates increased abundance compared to the reference population. The details in score calculation for samples and taxa are proprietary and not available in the public domain. The GA-map dysbiosis test was developed based on samples from 668 adults, including healthy controls (*n* = 297), patients with irritable bowel syndrome (IBS) (*n* = 236), and patients with inflammatory bowel disease (IBD) (*n* = 135). The test has been validated in independent cohorts and showed proportions of dysbiosis of 73% in IBS, 70% in treatment-naive IBD, 80% in IBD in remission, and 16% in healthy individuals (16).

### Generalizations and extensions.

The GA-map dysbiosis test, outlined above, was originally developed and validated for diagnosis and prediction of IBD and IBS; however, studies have also applied the test to evaluate its correlation with the effect of various interventions, including fecal microbiota transplantation, or FMT (17–19), dietary interventions (20–22), and anti-tumor necrosis factor (anti-TNF) therapy against ulcerative colitis, or UC (23). The GA-map index score was reportedly reduced following FMT (18, 19), and this was further lowered by repetitive FMT (17). The index was additionally reported to capture the response of the gut microbiota to a diet low in fermentable oligosaccharides, disaccharides, monosaccharides, and polyols (FODMAP), which, notably, led to an increase of the dysbiosis index (17, 21). However, other reports find that a low FODMAP diet did not have any effect (22). Magnusson et al. (23) showed that the effect of anti-TNF therapy for patients with UC was partially determined by the gut microbiota composition before treatment and that nonresponders had a higher GA-map index than responders. Another study assessed the performance of the GA-map dysbiosis index for detection of IBS when morbid obesity was considered (24). Four groups of subjects with or without IBS and with or without morbid obesity were compared. Dysbiosis was more frequently detected in morbidly obese subjects, regardless of IBS conditions, than in healthy volunteers. This underlines that confounding factors should be considered when using the GA-map dysbiosis test.

The GA-map dysbiosis test has also been applied to assess the effect of weight loss interventions followed by bariatric surgery (25), nonnutritive sweeteners, or NNSs (26), and primary Sjögren’s syndrome, or pSS (27). The dysbiosis index increased after weight loss interventions followed by bariatric surgery and was positively associated with the intake of NNSs. Dysbiosis was additionally found to be more prevalent in pSS patients than in healthy controls.

Some indexes were adapted for specific applications from the GA-map dysbiosis test (13, 22). Farup et al. (13) adjusted the GA-map dysbiosis test and thereby created a new index, named alternative index (ADI) (index 1.2), which allowed differentiation between microbiota disturbances caused by metformin, which has antihyperglycemic and weight-reducing effects (28, 29), and microbiota disturbances caused by NNSs, which may induce glucose intolerance (30). While the ADI showed opposite responses to these two interventions, the unmodified GA-map dysbiosis test yielded increased scores in both cases.

For quantification and prediction of the response of IBS patients to a 4-week FODMAP-restricted diet (22), a specific response index (RI) based on the GA-map dysbiosis test was created (index 1.3). Response was defined as a reduction in IBS severity scores of greater than 50%. First, the responders’ median bacterial scores (derived from the GA-map dysbiosis test) of 10 selected taxa, which differed between responders and nonresponders, were determined and defined as the cutoff. Each sample was then given a point for each taxon if the less abundant taxa (responders versus nonresponders) had lower scores than the cutoff or if the more abundant taxa (responders versus nonresponders) had higher scores than the cutoff. The summed points for each sample resulted in an RI range between 1 and 10. Thus, RI was more sensitive than the GA-map dysbiosis test to distinguish and predict treatment outcomes, since it was designed to consider only taxa differing between responders and nonresponders.

### Category 2: relevant taxon-based methods.

A large number of studies have utilized relevant taxa to create dysbiosis indexes. Such indexes only require the abundances of specific taxa and have been widely used in studies due to their simplicity, especially when sequencing data are available. They are easy to interpret and visualize and are typically calculated based on ratios between abundances, differences between abundances, or abundance-based linear regressions. The dysbiosis indexes introduced here are all based on relative abundances; however, other types of normalizations are also possible (31).

Among these approaches, the method of Gevers et al. was the first introduced and is currently the most widely used (32) (index 2.1). It is known that an altered gut microbial community composition is associated with the pathogenesis of IBD (33). To quantify and define the associations between gut microbiota and Crohn’s disease (CD), Gevers et al. compared ileum, rectum, and stool microbiotas from a large number of subjects with CD (*n* = 447) with those from healthy controls (*n* = 221). An overall decrease in richness as well as an altered gut microbiota composition was observed in CD subjects. One set of genera was positively correlated with CD, while another set of genera was negatively correlated. Based on this, the authors developed a dysbiosis index (index 2.1), defined as

$${\text{log}}_{e}\frac{\sum_{i=1}^{n}{\text{abundance(CD-enriched taxon)}}_{i}}{\sum_{i=1}^{n}{\text{abundance(CD-depleted taxon)}}_{i}}$$

This index was found to be negatively associated with species richness and positively associated with CD severity.

### Generalizations, extensions, and variants.

Gevers’ dysbiosis index was later modified for detection of other diseases and conditions. In a study of cirrhosis, Bajaj et al. (34) used the ratio between summed relative abundance of taxa, which in previous studies had been found to be reduced in cirrhosis patients (*Lachnospiraceae*, *Ruminococcaceae*, and *Clostridiales* XIV), and summed relative abundances of previously identified cirrhosis-associated taxa (*Enterobacteriaceae* and *Bacteroidaceae*) (index 2.2):

$$\frac{\sum_{i=1}^{n}{\text{abundance(health-related taxon)}}_{i}}{\sum_{i=1}^{n}{\text{abundance(patient-related taxon)}}_{i}}$$

Low index values indicated dysbiosis. The index was observed to be higher in healthy controls than in cirrhosis patients and was negatively correlated with endotoxin, death, and organ failures.

In a study of chronic hepatitis B (CHB), Wang et al. (6) defined the dysbiosis index (index 2.3) as

$$\frac{\sum_{i=1}^{n}{\text{abundance(patient enriched taxon)}}_{i}}{n}-\frac{\sum_{i=1}^{n}{\text{abundance(health enriched taxon)}}_{i}}{n}$$

Thus, this type of index uses the normalized abundance difference instead of the ratio between abundances to quantify dysbiosis. *n* refers to the number of taxa enriched in either the diseased or healthy state. The larger the index value, the more severe the dysbiosis. Thereafter, the authors applied Youden’s J statistic to look for the optimal index value as a diagnostic threshold to differentiate healthy from CHB patients. The cutoff was set at −25.36, thereby achieving 0.77, 0.75, and 0.81 for accuracy, sensitivity, and specificity, respectively.

Similar indexes include a multiplication by 100 or even by 10^6^. For example, Xia et al. (35) defined a dysbiosis index for acute ischemic stroke (index 2.4) as

$$[\frac{\sum_{i=1}^{n}\text{abundance}{\left(\text{patient enriched taxon}\right)}_{i}}{n}-\frac{\sum_{i=1}^{n}\text{abundance}{\left(\text{health enriched taxon}\right)}_{i}}{n}]\times 100$$

The index was calculated based on 18 discriminant taxa, and it achieved AUCs (area under the receiver operating characteristic curves) of 0.749 in the training cohort and 0.843 in the validation cohort, which indicates a good power for differentiating stroke patients from controls. Patients with stroke showed significantly higher index values than healthy controls.

Along the same lines, a study of patients with gout (36) defined a microbial index of gout (index 2.5) as

$$(\frac{\sum_{i=1}^{n}\text{abundance}{\left(\text{patient-enriched taxon}\right)}_{i}}{n}-\frac{\sum_{i=1}^{n}\text{abundance}{\left(\text{health-enriched taxon}\right)}_{i}}{n})\times 10^{6}$$

The index threshold was set at −2.157 by use of Youden’s J statistic. An index above the threshold indicated increased risk of suffering from gout. This index achieved an AUC of 0.817 for the identification of individuals diagnosed with gout.

Some reports define more complicated dysbiosis indexes. To have an index to describe the tumor burden in colorectal cancer development, Nakatsu et al. (5) devised a composite index including the microbiota at multiple sample sites, such as carcinoma (case 1), carcinoma-adjacent (case 2), adenoma (case 3), and adenoma-adjacent (case 4). The dysbiosis index (index 2.6) was then formulated to incorporate the differences in all cases:

$${\text{log}}_{10}[\left(\frac{\sum_{i=1}^{n}\text{abundance (enriched taxon in case 1}{)}_{i}}{\sum_{i=1}^{n}\text{abundance (depleted taxon in case 1}{)}_{i}}\times \frac{\sum_{i=1}^{n}\text{abundance (enriched taxon in case 2}{)}_{i}}{\sum_{i=1}^{n}\text{abundance (depleted taxon in case 2}{)}_{i}}\times \frac{\sum_{i=1}^{n}\text{abundance (enriched taxon in case 3}{)}_{i}}{\sum_{i=1}^{n}\text{abundance (depleted taxon in case 3}{)}_{i}}\times \frac{\sum_{i=1}^{n}\text{abundance (enriched taxon in case 4}{)}_{i}}{\sum_{i=1}^{n}\text{abundance (depleted taxon in case 4}{)}_{i}}\right)+1]$$

In contrast to these complex approaches, some studies defined a dysbiosis index based on only a very few taxa. For example, a study included only two relevant taxa in a linear regression to create a dysbiosis index (10). Two relevant taxa found to be significantly associated with recurrent aphthous stomatitis (RAS) (Acinetobacter johnsonii) and absence of RAS (Streptococcus salivarius), respectively, were identified based on logistic regression. The coefficients of these two species were then incorporated in a linear regression, and the dysbiosis index was defined as 5.35 × [*A. johnsonii*] − 0.309 × [*S. salivarius*], using the relative abundance of *A. johnsonii* and *S. salivarius* in the mucosa (index 2.7). This dysbiosis index correctly predicted 83% of the total cases for the presence or absence of RAS in the investigated cohort.

Even simpler, some studies used the ratio of only two phyla, such as *Firmicutes* and *Bacteroidetes*, to calculate an index for the description of the gut microbiota (index 2.8). Together, these two phyla constitute the majority of the human gut bacteria, and they represent the Gram-positive and the Gram-negative populations, respectively. Jeffery et al. (37) applied the *Firmicutes*-to-*Bacteroidetes* (F/B) ratio and successfully differentiated two subgroups (F/B high and F/B low) of patients with IBS. Stratification into high and low F/B ratios allowed the identification of different gut microbiotas in IBS versus healthy controls, which was only found in the high F/B group. Liu et al. (38) applied the F/B ratio for studies of patients with liver cirrhosis and observed higher ratio values in patients than in healthy controls. In patients with heart failure (HF), the microbiota was characterized by a decreased F/B ratio and a reduced bacterial diversity, which was associated with clinical outcome (39). In addition, F/B ratio has been linked with obesity; however, this is still controversial (40, 41).

While most gut dysbiosis indexes are based on the bacterial community, Sokol et al. (42) defined the index as the abundance ratio between the two fungal phyla *Basidiomycota* and *Ascomycota*, since these two phyla showed differential abundances across phenotypes classified as IBD, IBD with flare (IBDf), IBD in remission (IBDr), and healthy controls. These two fungal phyla additionally exhibited a strong inverse correlation to each other (index 2.9). The index distinctly separated samples originating from different phenotypes, as healthy subjects scored significantly lower values than IBD, IBDf, and IBDr subjects and IBDf subjects scored significantly higher values than IBDr subjects.

### Category 3: neighborhood classification.

Neighborhood classification is a way to quantify the deviation of samples from a reference sample set based on the microbial composition assessed with the distance or dissimilarity matrices. Hence, it has been used as a measurement of microbial dysbiosis in given individuals compared to a population of healthy controls. By its application, Lloyd-Price et al. (43) defined a dysbiosis score as the median value of Bray-Curtis dissimilarity between the test sample and a healthy reference population (non-IBD metagenomes) (index 3.1). This index was developed from either the taxonomic or the metabolomic composition of a sample with the computational tools MetaPhlAn2 and HUMAnN2, respectively (44, 45). The tools include internal normalization to the total pool of taxa or metabolites. To determine whether a sample was dysbiotic, a threshold was defined as the 90th percentile of the dysbiosis score in the non-IBD samples. Hence, the median distance value of a test sample above the threshold indicates dysbiosis. This dysbiosis index was observed to distribute differently across disease phenotypes (non-IBD, UC, and CD).

In a more complicated way, AlShawaqfeh et al. (46) introduced a dysbiosis index for dogs with chronic inflammatory enteropathy (CE) (index 3.2), defined as

(Euclidean distance between the test sample and the healthy class centroid)−(Euclidean distance between the test sample and the diseased class centroid)

Thus, the index expresses the difference between the distances to the average healthy and diseased populations for a given microbiota sample. To be disease specific, the Euclidean distance was calculated based on quantification by quantitative PCR (qPCR) of seven carefully selected taxa that were shown to be significantly correlated with CE. An index of zero indicated that the sample had an equal distance to the center of both (healthy and diseased) populations. An index above zero designated deviation from the normal healthy state. This index achieved 74% sensitivity and 95% specificity for separation of healthy and CE dogs. Due to this good performance, numerous following studies utilized this index to quantify microbial dysbiosis for dogs or cats with CE (47, 48), response to food (49, 50), anthelmintic treatment (51), FMT (52), and alterations after intensive physical activity (53).

Montassier et al. (54) introduced a test named “CLOUD” to look for outliers within a given set of microbiota samples. Although the test was not directly intended to be a measure of dysbiosis, the log_2_-transformed CLOUD statistic was used as a dysbiosis score (55) (index 3.3). The normalization of taxon abundances depends on the distance matrix used to determine the CLOUD statistic. Here, normalization based on geometric means of pairwise ratios (GMPR) was used (56). A sample was considered dysbiotic if the CLOUD distance between the test sample and the healthy reference set was more than two standard deviations (SD) larger than the CLOUD distance mean within the healthy reference set. The CLOUD is a nonparametric test and makes no assumptions about the distribution of the reference sample set; thus, it may prove useful to identify less healthy microbial signatures under different conditions, diseases compared to healthy controls, or assessment of the restoration of microbiota following FMT.

### Category 4: random forest prediction.

The output from the machine learning algorithm random forest (out-of-bag probability, an internal estimation of prediction performance for samples left out of the bootstrap), fed with microbiota data from patients suffering from small intestinal bacterial overgrowth (SIBO) and healthy controls, has been suggested as a dysbiosis index (designated “symptom index”) (55) (index 4). This index was based on the operational taxonomic unit (OTU) abundances normalized by GMPR (56). This index ranges from 0 to 1, where values approaching 1 indicate high likelihood of the gut microbiota coming from symptomatic patients. The index successfully differentiated patients with SIBO from healthy controls (AUC, 0.896). Additionally, the index was observed to be associated with specific patient characteristics, such as age and antibiotics use.

### Category 5: combined alpha and beta diversity.

Alpha and beta diversity have routinely been used in sequencing-based microbiota studies and provide a general description of microbial communities. Alpha diversity, applied for describing the amount of unique taxa (richness) and their distribution (evenness) within a microbial community, is often considered a biomarker of health, since a low gut bacterial alpha diversity in adults is known to be associated with risk markers related to metabolic health (57, 58). Beta diversity, used for assessing differences in microbial community composition between individuals, is also commonly applied to assess differences between patients and healthy controls. Recently, a study combined alpha (Shannon index) and beta diversity (Jensen Shannon divergence, or JSD) and created a dysbiosis index for patients with Clostridioides difficile treated with FMT (1). The dysbiosis index of a sample (index 5) was defined as

(average difference in Shannon index between the test sample and each of the donors)×(average JSD between the test sample and each of the donors)

This index generally ranged from 0 to 5. Healthy controls usually have an index between 0 and 1, and patients with dysbiosis have index values larger than 1. This index achieved an AUC of 0.922 at differentiating pre-FMT samples from post-FMT samples.

## APPLICATIONS OF DYSBIOSIS INDEXES

Here, we reviewed existing methods to determine and quantify dysbiosis, including large-scale bacterial marker profiling, relevant taxon-based methods, neighborhood classification, random forest prediction, and combined alpha and beta diversity. These approaches all successfully captured the differences between microbiota related to specific conditions of disease or intervention and those present in healthy patients or at baseline (before an intervention).

The large-scale bacterial marker profiling measures a large group of bacterial markers to assess the divergence of a sample from the healthy controls. The GA-map dysbiosis test was specifically designed to diagnose IBD and IBS and has been widely used. However, confounding factors, such as obesity, need to be considered when using this test to avoid misleading conclusions (24). The indexes 1.2 and 1.3 represent modifications of the GA-map dysbiosis test and show the potential to reformulate the basic GA-map dysbiosis test scores for specific purposes.

The relevant taxon-based methods were also developed to be disease specific. Because the relevant taxa can be readily identified under any deviating conditions, they can easily be adapted to different diseases or conditions. Relevant taxon-based methods are convenient dysbiosis index alternatives when next-generation sequencing data are available, such as 16S rRNA gene amplicon sequences or shotgun metagenomic sequences. While the relevant taxon-based dysbiosis index 2.1 was developed in a large sample set, subsequently validated in a different cohort of patients with CD, and prevalently used in other studies, the remaining indexes were much less validated and often developed and utilized to explain the same data set upon which they were based. Thus, the specifically chosen relevant taxa may not be valid in other studies due to the differences in sequencing techniques, statistical analysis, individual differences, and confounding factors.

The neighborhood classification methods utilize distance or dissimilarity matrices to quantify if a test sample is significantly different from a set of healthy controls. Index 3.1 is a simple way of using the distance matrices by subjectively choosing a threshold of distance to distinguish between dysbiotic and nondysbiotic samples. Index 3.2 assesses the closeness of a test sample to the groups of diseased and healthy samples, respectively, in a more complicated way. Because the distance matrix is based on seven carefully selected taxa, index 3.2 is restricted to the disease CE, but generalizing this approach to other conditions remains a possibility. Index 3.3 is a robust, nonparametric outlier test, which makes it an appropriate index for many different conditions.

Random forest is a popular choice for researchers that use machine learning techniques for large and complex biological data sets (59), such as gut microbiota sequence data. Index 4 uses the original out-of-bag probability from random forest as the dysbiosis index to quantify the similarity between a test sample and the dysbiotic samples. This index is not restricted to a specific disease and can be used to differentiate dysbiotic samples from healthy controls. However, index 4 basically is the same as a binary classification of samples by random forest and does not provide much additional information, although the continuous index value potentially can be correlated with clinical characteristics (55).

The combined alpha and beta diversity takes advantage of the commonly used alpha and beta diversity and quantifies the difference between samples originating from patients and from donors of FMT. More validations are needed, because the definitions of dysbiosis and nondysbiosis are not clearly defined in the study.

## CONCLUSIONS

Several dysbiosis indexes have been successfully applied to characterize the gut microbiota in patients with different diseases or conditions. They may have important applications in the context of given diseases and treatments.

However, it is important to emphasize that the existence of dysbiosis as measured by a specific index does not imply that the dysbiosis is in any way causal to the given disease. In fact, the altered microbiota characterizing a given disease or intervention often results from alterations in factors such as diet, medication, oxygen availability, or immune reactions (14, 60–63), in which case a dysbiosis index is applicable as a diagnostic marker but not necessarily as a predictor. Difficulties in inferring general principles for the assessment of dysbiosis are, to a large extent, attributed to the huge variation between healthy individuals, leading to a lack of a clear definition of a “normal” gut microbiota. In fact, the existence of a balance of the microbiota that can suddenly tip over remains to be proven and is highly debated (12). It is important to note that dysbiosis indexes are not standalone measurements and have to be interpreted in the context of the clinical findings. Nevertheless, the value of dysbiosis indexes as simple tools to describe complex differences between intestinal microbial communities remains.
